# Supplementary material for: Mechanisms governing the pioneering and redistribution capabilities of the non-classical pioneer PU.1
Source: Nat Commun. 2020 Jan 21;11:402. doi: 10.1038/s41467-019-13960-2 (PMC6972792; doi:10.1038/s41467-019-13960-2)
Supplement: Supplementary file 7 — Source data [file 41467_2019_13960_MOESM7_ESM.zip › Source_Data/FigureS3/FigureS3H_MotifScanOutput/homerResults/motif6.similar.html]

motif6

## Information for motif6

T
G
C
A
G
C
T
A
T
C
A
G
A
G
T
C
C
G
T
A
A
T
C
G
A
T
G
C
C
G
A
T
A
C
T
G
A
G
T
C
C
A
G
T
  
Reverse Opposite:  

G
T
C
A
T
C
A
G
A
G
T
C
C
G
T
A
A
T
C
G
A
T
G
C
C
G
A
T
A
C
T
G
A
G
T
C
C
G
A
T
A
C
G
T
  

|  |  |
| --- | --- |
| p-value: | 1e-130 |
| log p-value: | -3.006e+02 |
| Information Content per bp: | 1.664 |
| Number of Target Sequences with motif | 939.0 |
| Percentage of Target Sequences with motif | 24.29% |
| Number of Background Sequences with motif | 4452.8 |
| Percentage of Background Sequences with motif | 9.94% |
| Average Position of motif in Targets | 291.3 +/- 267.0bp |
| Average Position of motif in Background | 210.5 +/- 151.3bp |
| Strand Bias (log2 ratio + to - strand density) | 0.0 |
| Multiplicity (# of sites on avg that occur together) | 1.20 |
| Motif File: | file (matrix) reverse opposite |

### Similar de novo motifs found

|  |  |  |  |  |  |  |  |
| --- | --- | --- | --- | --- | --- | --- | --- |
| Rank | Match Score | Redundant Motif | P-value | log P-value | % of Targets | % of Background | Motif file |
| 1 | 0.972 | G T A C T C G A T C A G A G T C C G T A A T C G A T G C C G A T A C T G G A T C | 1e-108 | -249.030473 | 34.14% | 18.40% | motif file (matrix) |
| 2 | 0.954 | G T C A T C A G A T G C C G T A T C A G A T G C G C A T T A C G | 1e-106 | -244.942410 | 36.19% | 20.17% | motif file (matrix) |
| 3 | 0.944 | A G T C C G T A A C T G A G T C A C G T A C T G A G T C | 1e-101 | -234.770960 | 34.51% | 19.09% | motif file (matrix) |
| 4 | 0.978 | T C G A T C A G A T G C C G T A T A C G A T G C C G A T A C T G A G T C C G A T A C T G G A T C | 1e-100 | -231.390846 | 39.19% | 23.07% | motif file (matrix) |
| 5 | 0.961 | T C G A G T A C C G T A T A C G A T G C G C A T A C T G A G C T A C G T | 1e-96 | -221.253679 | 30.26% | 16.10% | motif file (matrix) |
| 6 | 0.912 | T C A G G T A C G C T A T A C G A T G C C G A T A C T G A T G C G C A T T A C G G A T C C A G T A C T G | 1e-90 | -209.520865 | 27.86% | 14.55% | motif file (matrix) |
| 7 | 0.822 | G C T A T C A G A T G C C G T A C T A G A C T G C A G T A C T G A T G C | 1e-51 | -117.888913 | 20.15% | 11.30% | motif file (matrix) |
| 8 | 0.767 | T G A C C T G A T A C G G T A C C G A T A C T G A C G T G C A T | 1e-33 | -76.325328 | 14.61% | 8.41% | motif file (matrix) |
